# Non-vitamin K oral anticoagulants are non-inferior for stroke prevention but cause fewer major bleedings than well-managed warfarin: A retrospective register study

**DOI:** 10.1371/journal.pone.0181000

**Published:** 2017-07-10

**Authors:** Vilhelm Sjögren, Björn Byström, Henrik Renlund, Peter J. Svensson, Jonas Oldgren, Bo Norrving, Anders Själander

**Affiliations:** 1 Department of Public Health and Clinical Medicine, Umeå University, Umeå, Sweden; 2 Uppsala Clinical Research Centre, Uppsala University, Uppsala, Sweden; 3 Department of Translational Medicine, Clinical Coagulation Research Unit, Skåne University Hospital, Lund University, Malmö, Sweden; 4 Uppsala Clinical Research Centre and Department of Medical Sciences, Uppsala University, Uppsala, Sweden; 5 Department of Clinical Sciences Lund, Neurology, Skåne University Hospital, Lund University, Lund, Sweden; Institut d'Investigacions Biomediques de Barcelona, SPAIN

## Abstract

**Background:**

For patients with atrial fibrillation, non-vitamin K oral anticoagulants, or NOACs (dabigatran, rivaroxaban, edoxaban, and apixaban) have been proven non-inferior or superior to warfarin in preventing stroke and systemic embolism, and in risk of haemorrhage. In the pivotal NOAC studies, quality of warfarin treatment was poor with mean time in therapeutic range (TTR) 55–65%, compared with ≥70% in Swedish clinical practice.

**Methods:**

We compared NOACs (as a group) to warfarin in non-valvular atrial fibrillation, studying all 12,694 patients starting NOAC treatment within the Swedish clinical register and dosing system Auricula, from July 1, 2011 to December 31, 2014, and matching them to 36,317 patients starting warfarin using propensity scoring. Endpoints were thromboembolic events and major bleedings that were fatal or required hospital care. Outcome data were collected from validated Swedish hospital administrative and clinical registers.

**Results:**

Mean age was 72.2 vs 72.3 years, proportion of males 58.2% vs 57.0%, and mean follow-up time 299 vs 283 days for NOACs and warfarin. Distribution of NOACs was: dabigatran 40.3%, rivaroxaban 31.2%, and apixaban 28.5%. Mean TTR was 70%. There were no significant differences in rates of thromboembolic/thrombotic events or gastrointestinal bleeding. NOAC treated patients had lower rates of major bleeding overall, hazard ratio 0.78 (95% confidence interval 0.67–0.92), intracranial bleeding 0.59 (0.40–0.87), haemorrhagic stroke 0.49 (0.28–0.86), and other major bleeding 0.71 (0.57–0.89).

**Conclusion:**

For patients with atrial fibrillation, NOACs are as effective for stroke prevention as well-managed warfarin but cause fewer major bleedings.

## Introduction

Atrial fibrillation is a strong risk factor for ischaemic stroke. Anticoagulation with vitamin K antagonists, e.g. warfarin, reduces this risk by about two-thirds and mortality by one quarter, but increases the risk of haemorrhage compared to no treatment.[1] Warfarin has been recommended for patients with atrial fibrillation in major guidelines for many years. Since 2011 several non-vitamin K antagonist oral anticoagulants, or NOACs, have been available in clinical practice in Sweden for prevention of stroke and systemic embolism in patients with atrial fibrillation. The term NOAC is used for the direct thrombin inhibitor dabigatran, as well as for direct factor Xa inhibitors such as rivaroxaban, apixaban, and edoxaban. In pivotal studies, NOACs have been proven superior or non-inferior to warfarin for both stroke prevention and risk of haemorrhage.[2–5] In a large Danish retrospective study, NOACs were confirmed to be at least as effective and safe as warfarin, but without data on the treatment quality of warfarin.[6]

The quality of warfarin treatment can be measured by frequencies of hard outcomes like haemorrhagic complications and non-prevented thromboembolic events. Another indicator of treatment quality is time in therapeutic range (TTR) i.e. the proportion of time that the patients’ anticoagulation is within the therapeutic range of International Normalized Ratio (INR) 2–3.[7] High TTR correlates to lower risk of both major bleeding and thromboembolic events.[8] In the pivotal NOAC trials, mean TTR in the warfarin treated control groups ranged from 55% to 65%. This is low compared to Swedish clinical practice. A recent Swedish publication of 51,299 patients with atrial fibrillation, with a total of 142,626 treatment years on warfarin found a TTR of 77.4%.[9] Another Swedish study of 40,449 patients with atrial fibrillation (65,424 treatment years) found TTR to be 68.6% for patients starting warfarin treatment.[10] A retrospective U.S. study of patients with atrial fibrillation, identified through healthcare insurance claims, found NOACs to be superior or equivalent to warfarin. TTR data were available only for a minority of patients, with median TTR 56%.[11] The potential benefit of NOACs over warfarin with high TTR has been questioned.[12, 13]

This study aimed at elucidating the efficacy and safety of NOACs compared to warfarin in patients starting oral anticoagulation due to non-valvular atrial fibrillation in a Swedish setting where TTR has repeatedly been shown to be high.

## Materials and methods

### Study design

We performed a retrospective cohort study comparing a group of the three NOACs that were licensed during the study period (apixaban, dabigatran, and rivaroxaban) to warfarin, in patients with atrial fibrillation within the Swedish national quality register Auricula.

### Ethical considerations

The study was approved by the Ethics review board in Umeå, 2015/142-31.

### Materials

Auricula is a system for warfarin dosing, monitoring and quality control of oral anticoagulation. It is utilized in 11 of Sweden’s 21 healthcare regions covering close to one-half of all patients on oral anticoagulation in Sweden, with 21% of the patients managed in primary care.[14] In general, whole Swedish health care regions join Auricula, and within those regions, all patients on oral anticoagulation are managed in Auricula, making patient selection unlikely. Monitoring of oral anticoagulation in clinical practice is performed within Auricula’s ordination system, with data automatically transferred to the quality register daily. Patients can choose not to be included in this quality monitoring. In 2015, Auricula contained information on more than 120,000 ordination (treatment) periods, of which about three-quarters are for stroke prevention in atrial fibrillation. Registered data include indication(s) for anticoagulation, start date, name and dose of drug, prothrombin time-INR values, temporary interruptions, and creatinine measurements for NOAC patients.

We merged Auricula with three other national clinical registers. The Swedish National Patient Register has data on all diagnoses as well as surgical and other procedure codes made at hospitals and specialist clinics, with a positive predictive value of diagnoses of 85–95%.[15] Patients’ medical backgrounds, as well as outcomes other than death and stroke, were extracted from this register. Diagnoses defining items in patients’ medical history are given in S1 Table. Since we used presence of a diagnosis as the definition of medical history, no grading was possible, e.g. of renal failure or heart failure. The Swedish Cause of Death Register covers all deaths in Sweden. The Swedish Stroke Register (Riksstroke) has high validity data with coverage of 96.5% on all cases of acute stroke from all hospitals in Sweden.[16]

### Study population

All patients within Auricula with the indication atrial fibrillation, excluding patients with mechanical heart valve prostheses, were selected. The first new anticoagulation treatment period between 1 July 2011 and 31 December 2014 was included. Switching from warfarin to NOAC treatment was allowed, but patients on continuous warfarin treatment on 1 July 2011 were excluded. Previous warfarin experience outside of Auricula was allowed, defined as any prescription of warfarin up until 30 days before start of the treatment period. Recent warfarin was defined as a prescription during the six months prior to 30 days before treatment.

### Procedures

We created a database by merging data from the registers described above, and a propensity score matching was performed as described below. After matching was performed, all members of the study group agreed to lock the database before outcome analyses commenced.

Diagrams showing the effect of matching on the distributions of age, treatment duration, and CHA_2_DS_2_-VASc score are given in S1–S3 Figs. Overall success of matching, measured as standardised difference, is described in S4 Fig. When interpreting the results, one should keep in mind that since we weighted the warfarin group to be equal in size to the NOAC group, the weighted number of events is not necessarily an integer. Outcome numbers are rounded in the presentation, but exact values were used for all analyses.

We calculated TTR based on all available individual INR values for the whole study period for all warfarin-treated patients, using Rosendaal’s method.[7]

### Definitions of outcomes

Outcomes were all-cause stroke and systemic embolism, all-cause mortality, all-cause stroke, ischaemic stroke, haemorrhagic stroke, myocardial infarction, and major bleeding events defined as intracranial, gastrointestinal, or other bleeding that was fatal or required hospital care. Data on acute ischaemic stroke and intracerebral haemorrhage were collected from Riksstroke, and on death from the Cause of Death Register. All other outcomes were extracted from the National Patient Register. Haemorrhagic stroke was defined as intracerebral haemorrhage or subarachnoid haemorrhage. Haemorrhagic stroke was an endpoint in itself, as well as a constituent of intracranial bleeding, all-cause stroke, and major bleeding.

Only the first occurrence of each type of endpoint counted, to reduce the risk of over-reporting. A patient could thus have one, two, or three types of bleeding (intracranial, gastrointestinal, and/or other) together with one or several types of thrombotic outcomes, as well as death.

Outcomes were similar to those used in the NOAC trials, but with the difference that we only classified outcomes that were fatal or required hospital care. Definitions of diagnoses qualifying as outcomes are given in S2 Table.

### Statistical analysis

Using logistic regression we estimated the probability of receiving a specific type of oral anticoagulant as treatment, i.e. the (estimated) propensity score, based on date of treatment start, age, sex, prior warfarin treatment as well as relevant diagnoses in patients’ medical history: myocardial infarction, stroke or TIA, hypertension, congestive heart failure, diabetes, vascular disease, liver disease, renal failure, excessive alcohol use, percutaneous coronary intervention, intracranial bleeding, gastrointestinal bleeding, and other bleeding. These scores were utilized in full optimal matching, which is an algorithm that creates variable size clusters, containing both cases (NOAC) and controls (warfarin), with similar propensity scores, such that the overall average discrepancies (the Euclidean distance) between the scores within clusters are minimized.[17–19] Each case is given a weight of one (1) and the controls are given weights such that every cluster is balanced. Thus we expect the weighted control population to resemble (in distribution) the case population on the covariates included in the propensity score.

Outcomes were regressed on treatment as well as the covariates used in the propensity score.[20, 21] More specifically, a weighted Cox proportional hazards model was used with robust variance ("sandwich") estimates. Analyses and graphics were done using the R software, version 3.3 (R Foundation for Statistical Computing, Vienna, Austria).[19, 22, 23]

## Results

### Baseline characteristics of the studied patients

A total of 49,011 patients were identified, 12,694 starting a NOAC and 36,317 starting warfarin. Distribution of NOACs was dabigatran 5,118 (40.3%), rivaroxaban 3,961 (31.2%), and apixaban 3,615 (28.5%). 41.1% in the NOAC group had a previous warfarin prescription, of which 16.8% were recent (a prescription during the six months prior to 30 days before treatment start). For warfarin treated patients, the rates were 34.8 and 18.2%.

Table 1 shows the demographics and relevant medical history for all patients on NOAC, and for all patients on warfarin before and after matching and weighting. The proportion of patients receiving NOAC treatment was only 1% in 2011 but increased to 12% in 2012, and equaled that of warfarin treatment in 2014.

**Table 1:** Background characteristics of study participants according to treatment, before and after matching and weighting.

Treatment	NOAC	Warfarin unmatched	Warfarin matched and weighted
Number	12694	36317	12694
*Demographics*			
Male	7386 (58.2)	21580 (59.4)	7254 (57.1)
Age	72.2 (10.3)	73.5 (10.2)	72.3 (10.3)
Duration (days)	299 (260)	504 (378)	283 (257)
Prior warfarin	5217 (41.1)	12647 (34.8)	5146 (40.5)
*Other*, *concomitant indication for oral anticoagulation*			
DC conversion	1382 (10.9)	6488 (17.9)	2149 (16.9)
Venous thromboembolism	174 (1.4)	848 (2.3)	364 (2.9)
Other indication	180 (1.4)	1100 (3.0)	359 (2.8)
*Medical history*			
History of fall	1922 (15.1)	4748 (13.1)	1870 (14.7)
Cancer	1615 (12.7)	4611 (12.7)	1549 (12.2)
Stroke	1713 (13.5)	5451 (15.0)	1852 (14.6)
Transient ischaemic attack (TIA)	903 (7.1)	2584 (7.1)	854 (6.7)
Stroke or TIA	2331 (18.4)	7223 (19.9)	2431 (19.2)
Hypertension	7809 (61.5)	21729 (59.8)	7822 (61.6)
Congestive heart failure	1504 (11.8)	4590 (12.6)	1447 (11.4)
Diabetes	1952 (15.4)	6414 (17.7)	2026 (16.0)
Myocardial infarction	1903 (15.0)	6550 (18.0)	1966 (15.5)
Chronic obstructive pulmonary disease	1451 (11.4)	4077 (11.2)	1431 (11.3)
Anemia	1099 (8.7)	3097 (8.5)	1127 (8.9)
Major bleeding	3161 (24.9)	7872 (21.7)	3106 (24.5)
Gastrointestinal bleeding	740 (5.8)	1788 (4.9)	726 (5.7)
Intracranial Bleeding	319 (2.5)	597 (1.6)	348 (2.7)
Cerebral haemorrhage	234 (1.8)	432 (1.2)	205 (1.6)
Previous traumatic intracranial bleeding	122 (1.0)	235 (0.6)	184 (1.5)
Other bleeding	2455 (19.3)	6220 (17.1)	2422 (19.1)
Renal failure	434 (3.4)	2255 (6.2)	431 (3.4)
Excessive alcohol use	381 (3.0)	852 (2.3)	368 (2.9)
Dementia	175 (1.4)	434 (1.2)	169 (1.3)
Liver disease	182 (1.4)	490 (1.3)	185 (1.5)
Vascular disease	2314 (18.2)	7785 (21.4)	2413 (19.0)
Percutaneous coronary intervention (PCI)	990 (7.8)	3099 (8.5)	1024 (8.1)
CHA_2_DS_2_-VASc	3.30 (1.85)	3.49 (1.88)	3.37 (1.87)

Frequencies are given as numbers and percentages. Age, duration, and CHA_2_DS_2_-VASc score are given as means with standard deviation.

History of fall was defined as at least two occurrences of a relevant ICD-10 code (S1 Table). CHA_2_DS_2_-VASc is a score of stroke risk, with 1 point each for congestive heart failure, hypertension, diabetes, vascular disease, and female sex. Stroke or TIA: 2 points. Age 65–74: 1 point, ≥75: 2 points.

### Baseline characteristics of the matched study patients

Baseline data are given for patients on NOACs and propensity matched and weighted controls on warfarin. Mean age was 72.2 for NOACs vs 72.3 years for warfarin. Proportion of males was 58.2% and 57.0%. Mean follow-up time was 299 vs 283 days. TTR was 70% during the study period for the matched warfarin treated patients, and 72% for the unmatched.

### Outcomes

**Fig 1:**
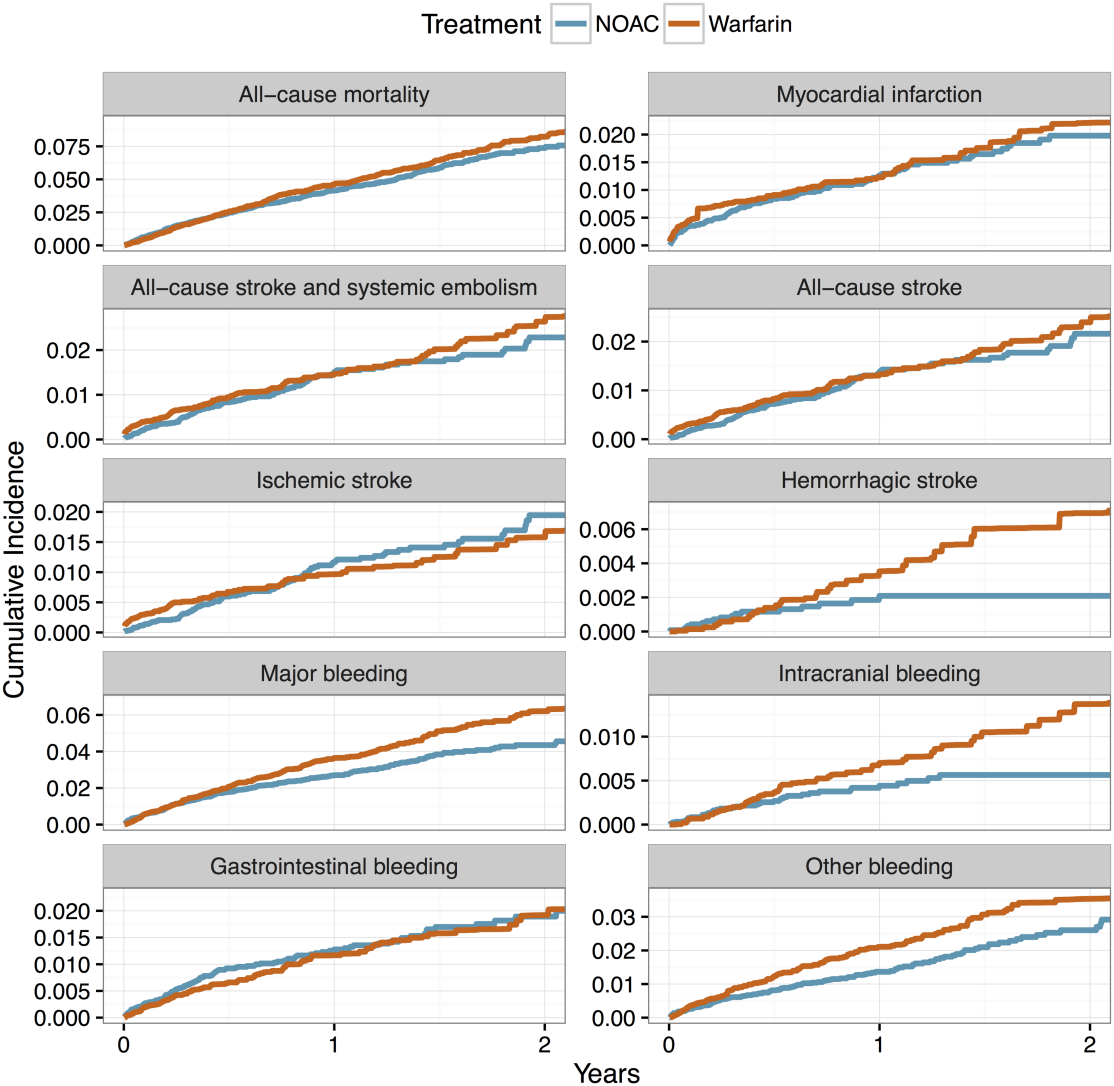
Incidence plots of endpoints.

Table 2 provides absolute numbers and annual rates of outcomes, and Fig 1 gives incidence plots of outcomes for patients on NOACs and matched patients on warfarin.

**Table 2:** Incidence rates and hazard ratios for outcomes according to treatment.

	NOACs		Matched warfarin			
	Number	Annual rate (%)	Number	Annual rate (%)	HR	p value
All-cause stroke and systemic embolism	139	1.35	154	1.58	0.89 (0.69–1.15)	0.36
All-cause stroke	125	1.21	137	1.40	0.89 (0.68–1.17)	0.41
Ischaemic stroke	107	1.04	101	1.03	1.04 (0.75–1.43)	0.83
Haemorrhagic stroke	17	0.16	35	0.35	0.49 (0.28–0.86)	0.01
Major bleeding	283	2.76	350	3.61	0.78 (0.67–0.92)	0.003
Intracranial bleeding	41	0.40	68	0.69	0.59 (0.40–0.87)	0.008
Gastrointestinal bleeding	130	1.26	112	1.14	1.14 (0.88–1.46)	0.32
Other bleeding	149	1.45	203	2.08	0.71 (0.57–0.89)	0.003
All-cause mortality	437	4.21	459	4.66	0.94 (0.82–1.07)	0.33
Myocardial infarction	129	1.25	142	1.45	0.95 (0.72–1.24)	0.68

Absolute numbers of outcomes and annual incidence rates (events/100 treatment years). Cox regression analysis of combined outcomes with hazard ratios (HR) with 95% confidence intervals. All-cause stroke is ischaemic stroke plus intracerebral and subarachnoidal haemorrhage. Haemorrhagic stroke is a component of all-cause stroke as well as intracranial bleeding.

Total numbers of deaths were 437 (annual rate 4.21%) for NOACs and 459 (4.66%) for warfarin. Stroke and systemic embolism occurred in 139 vs 154 cases (1.35% vs 1.58%), and all-cause stroke in 125 (1.21%) and 137 (1.40%). Strokes were mostly ischaemic 107 (1.04%) and 101 (1.03%).

Major bleeding occurred significantly less often with NOACs (283 cases corresponding to a 2.76% annual rate) than with warfarin (350 cases, 3.61%). There were 41 vs 68 intracranial haemorrhages, giving annual rates of 0.40% for NOACs vs 0.69% for warfarin, with 17 (0.16%) and 35 (0.35%) of those being intracerebral haemorrhages. Other major bleeding events were also significantly less frequent for patients treated with NOACs: 149 (1.45%) vs 203 (2.08%). Gastrointestinal bleeding was the only type of bleeding not to be less frequent with NOACs, with 130 cases (0.13%) in the NOAC group and 112 cases (0.11%) for warfarin (non-significant difference).

There were no significant differences in risk of all-cause stroke and systemic embolism, all-cause stroke, ischaemic stroke, and all-cause mortality.

**Fig 2:**
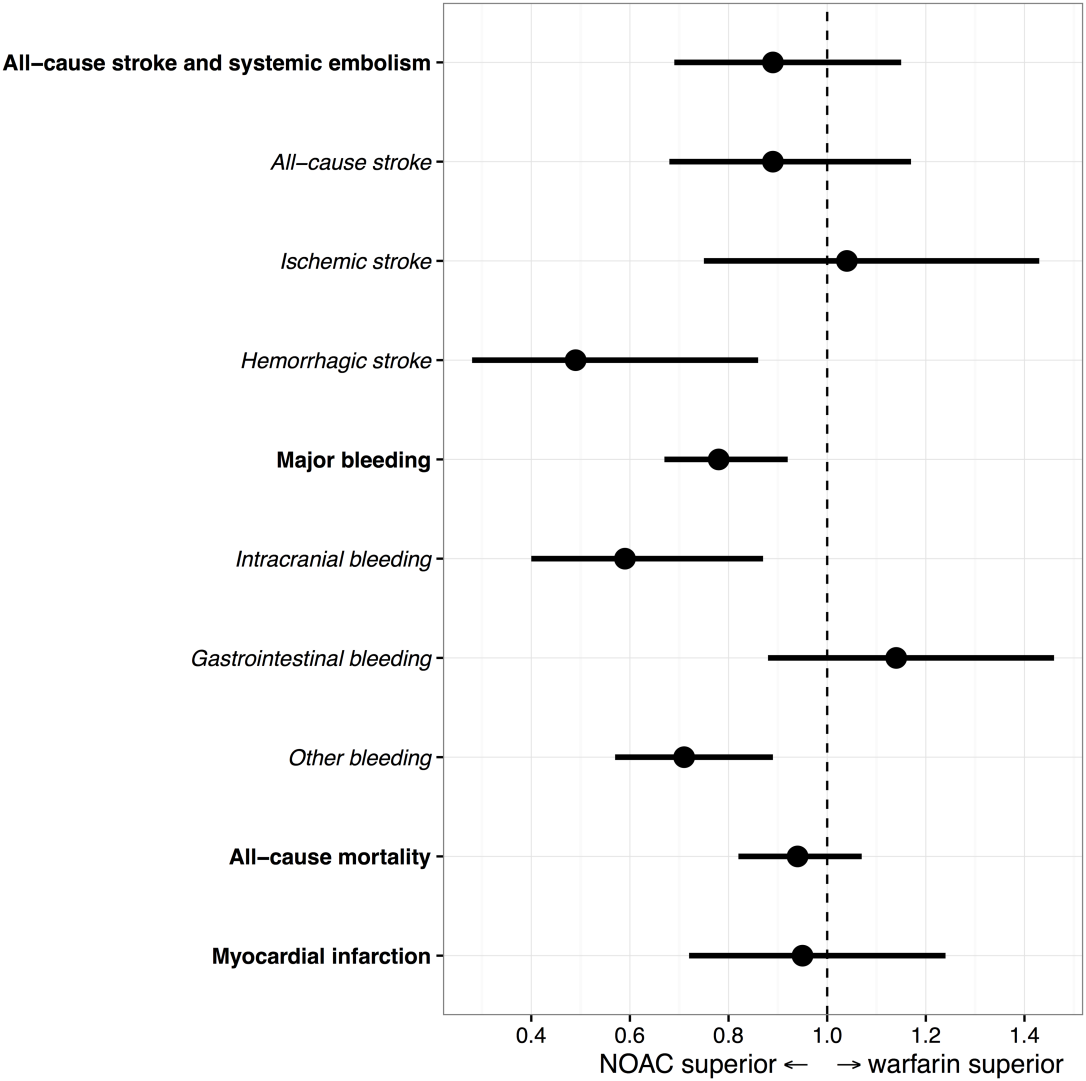
Forest plot of hazard ratios for outcomes. Point estimates and 95% confidence intervals.

Hazard ratios for outcomes are given in Table 2 and Fig 2.

## Discussion

In this large real-world register study of unselected patients with non-valvular atrial fibrillation starting oral anticoagulation for stroke prevention, we found that NOACs were not superior to warfarin, neither for preventing stroke or systemic embolism, nor for death, stroke, or myocardial infarction. This goes against a meta-analysis of the four pivotal NOAC trials where authors found a risk reduction of stroke and systemic embolism of 19%.[24] In that analysis, NOACs were favourable compared to warfarin with TTR both above and below 66%. Sub-group analyses of two of the NOAC trials indicated reduced benefit of NOACs with higher TTR.[12, 13] The TTR level of 70% in our study could account for some of the reduced benefit in efficacy of NOACs. It is a relatively high TTR for a cohort of newly started warfarin patients, but despite this, the risks of major bleeding, intracranial bleeding, haemorrhagic stroke, and other bleeding were significantly lower with NOACs than with warfarin. The high TTR level and the similar efficacy outcomes indicate that the advantage of NOACs in our study is not due to poor warfarin treatment.

Our main finding is that the risk of serious bleeding, particularly intracranial bleeding, is lower with NOACs than with warfarin, despite the warfarin treatment being well-managed by international standards, with an annual risk of intracerebral haemorrhage of 0.35%.[25] The annual rate of intracranial bleeding in our warfarin group of unselected real-world patients was 0.69%. This is in line with that of the selected warfarin patients in the pivotal NOAC trials (0.70% to 0.85%), but markedly higher than the 0.44% previously found in Swedish patients with atrial fibrillation starting warfarin treatment and followed for 1.6 years (0.34% if TTR≥70%, 0.72% if TTR<70%).[2–5, 10] Relatively short treatment duration and the fact that we sought for intracranial bleedings not only in the National Patient Register but also in the Cause of Death register, as well as in Riksstroke could at least partly explain the higher bleeding rate found in the present study. Intracranial bleeding rates over time differed between NOACs and warfarin, with incidence lines separating soon after treatment start, but one notices a flat incidence line for NOAC treated patients after one year. Relatively few patients started NOACs early in our study period and treatment time was therefore usually shorter than one year, so this would be expected. We have done what we could to achieve balance between treatment groups via matching, including matching for treatment start date. Nevertheless, we found a clear difference in favour of NOACs regarding risk of bleeding.

We have not analyzed the proportion of patients with ischaemic stroke just prior to starting anticoagulation. This group would be expected to have a higher risk of intracerebral haemorrhages than those with no recent stroke, and an imbalance could account for some of the difference in risk of haemorrhagic stroke.

The lack of difference in efficacy outcomes could be due to low numbers of events, but the rates of haemorrhagic complications resemble the findings in the randomised trials. The overall efficacy and safety of NOACs compared to warfarin seem to be confirmed in our study, despite a high TTR.

### Limitations

Since this is a retrospective cohort study, bias is to be expected. It is possible that the profile of patients that were prescribed NOACs and warfarin changed during the study period, with more cautious selection of patients starting NOACs in the first years. To overcome a possible imbalance between the treatment groups, we propensity matched patients starting warfarin or NOACs using start date as one variable. Residual confounding is both possible and likely. For example, even after matching, significantly more patients receiving warfarin had an additional indication for anticoagulation. This selection of patients with a less complicated background for NOAC treatment may have influenced our results, but would on the other hand have been expected to show a lower risk of thromboembolic and thrombotic events for NOACs, given the results of the randomised NOAC trials.

We did not exclude patients with prior experience of oral anticoagulation. However, in a large meta-analysis of the pivotal trials of NOACs versus warfarin, the benefit of NOACs seemed to be consistent regardless of prior anticoagulation experience.[24] Less than 20% of the patients in our study had a warfarin prescription during six months prior to 30 days before the treatment period that included them in the study. It seems unlikely that the results would change in any meaningful way if the analyses were done on the more than 80% of patients starting NOAC treatment that were anticoagulation-naïve. We therefore consider this to be a study mainly comparing patients starting oral anticoagulation with warfarin or a NOAC.

Comparisons between the three NOACs were deemed not meaningful due to limited numbers of patients.

The less strict control of NOAC treatment, as compared with the regular monitoring of warfarin, could mean worse compliance. This is a possibility in all studies not objectively controlling compliance. Bad compliance would confer lower bleeding risks, but should on the other hand mean relatively higher risks of ischaemic events, something that we did not find. Our results are in line with the randomised trials, with lower bleeding risks and similar risks of ischaemia.

The validity of the results from a register study depends on the quality of data. As for the quality of the registers we used, Riksstroke’s coverage is 90.3% when compared to the National Patient Register, which in turn has 6% over-reporting, meaning that the actual coverage is around 96%. Stroke diagnoses in the National Patient Register and the Cause of Death Register have a high validity.[26] To overcome the weaknesses of retrospective register studies, a prospective, randomised study comparing NOACs with well-managed warfarin would be preferred but has not been possible to fund, at least not in Sweden.

## Conclusion

For patients with non-valvular atrial fibrillation, oral anticoagulation with NOACs are as effective as well-managed warfarin for prevention of stroke and systemic embolism, but cause fewer major bleedings.

## Supporting information

S1 Fig Distribution of age before and after matching. Grouping in five-year clusters on the x-axis.
(TIFF)

S2 Fig Distribution of treatment duration before and after matching. The unit on the x-axis is days.
(TIFF)

S3 Fig Effect of matching on distribution of CHA_2_DS_2_-VASc score. Upper diagram before, lower diagram after matching. Note that the score was not part of the matching, although components were.
(TIFF)

S4 Fig Standardised difference for background variables between unmatched and matched populations. (TIFF)

S1 Table Definitions of medical history. Data source is the Swedish National Patient Register (NPR). ICD-10 codes for diagnoses. Other codes are Swedish procedure codes.
(DOCX)

S2 Table Definitions of outcomes. Data source is the Swedish National Patient Register (NPR) except for RS: The Swedish Stroke Register (Riksstroke), or death noted in the Cause of Death register.
(DOCX)

S1 Dataset Data on which the figures and tables in the article are based. (XLSX)

S2 Dataset Minimal data set. Anonymised data on all patients in the matched patient groups.
(XLSX)
