# Supplementary figures and images for: Non-vitamin K oral anticoagulants are non-inferior for stroke prevention but cause fewer major bleedings than well-managed warfarin: A retrospective register study
Source: PLoS One. 2017 Jul 10;12(7):e0181000. doi: 10.1371/journal.pone.0181000 (PMC5507293; doi:10.1371/journal.pone.0181000)

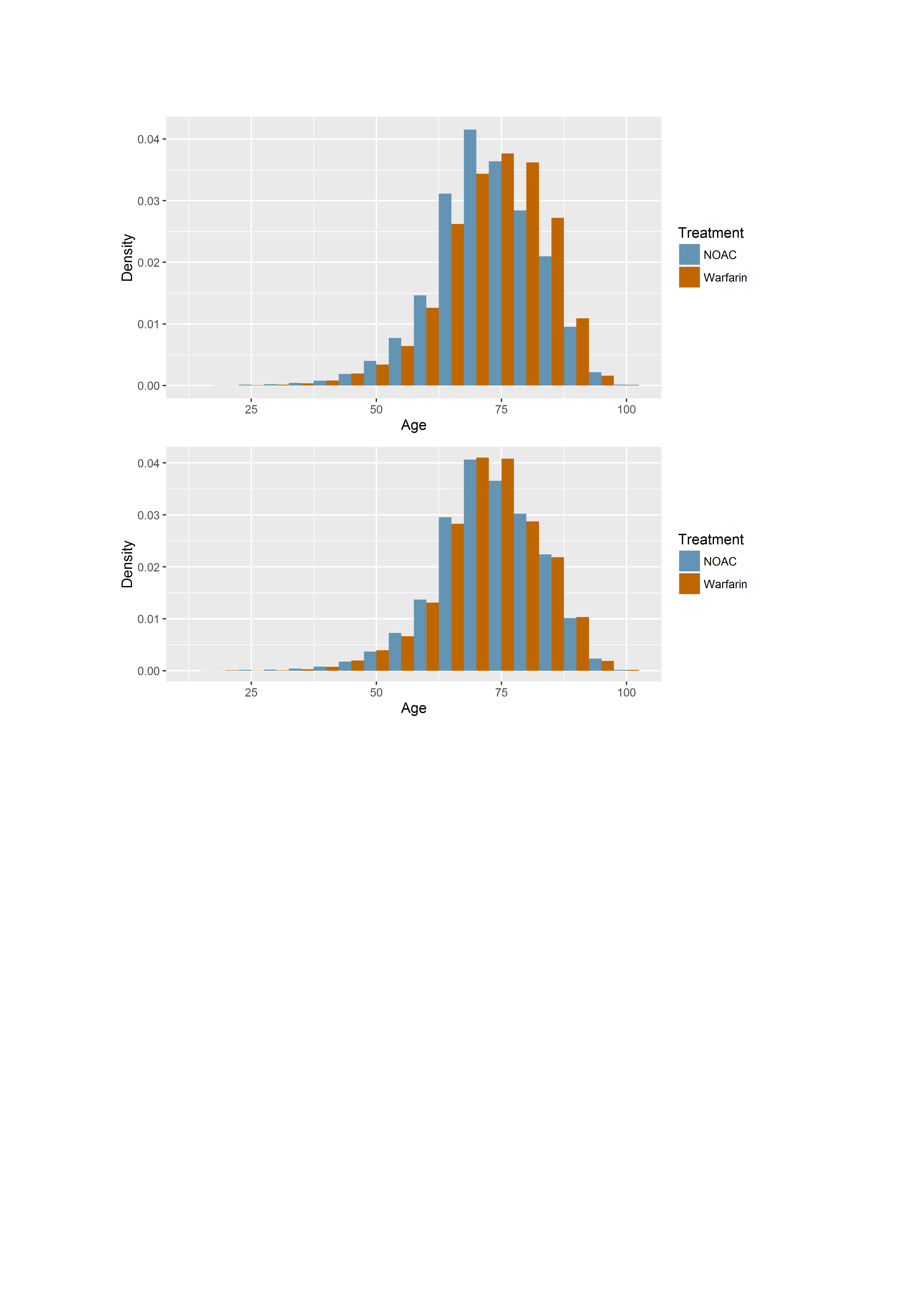

Supplement: S1 Fig — Grouping in five-year clusters on the x-axis. (TIFF) [file pone.0181000.s001.tiff]

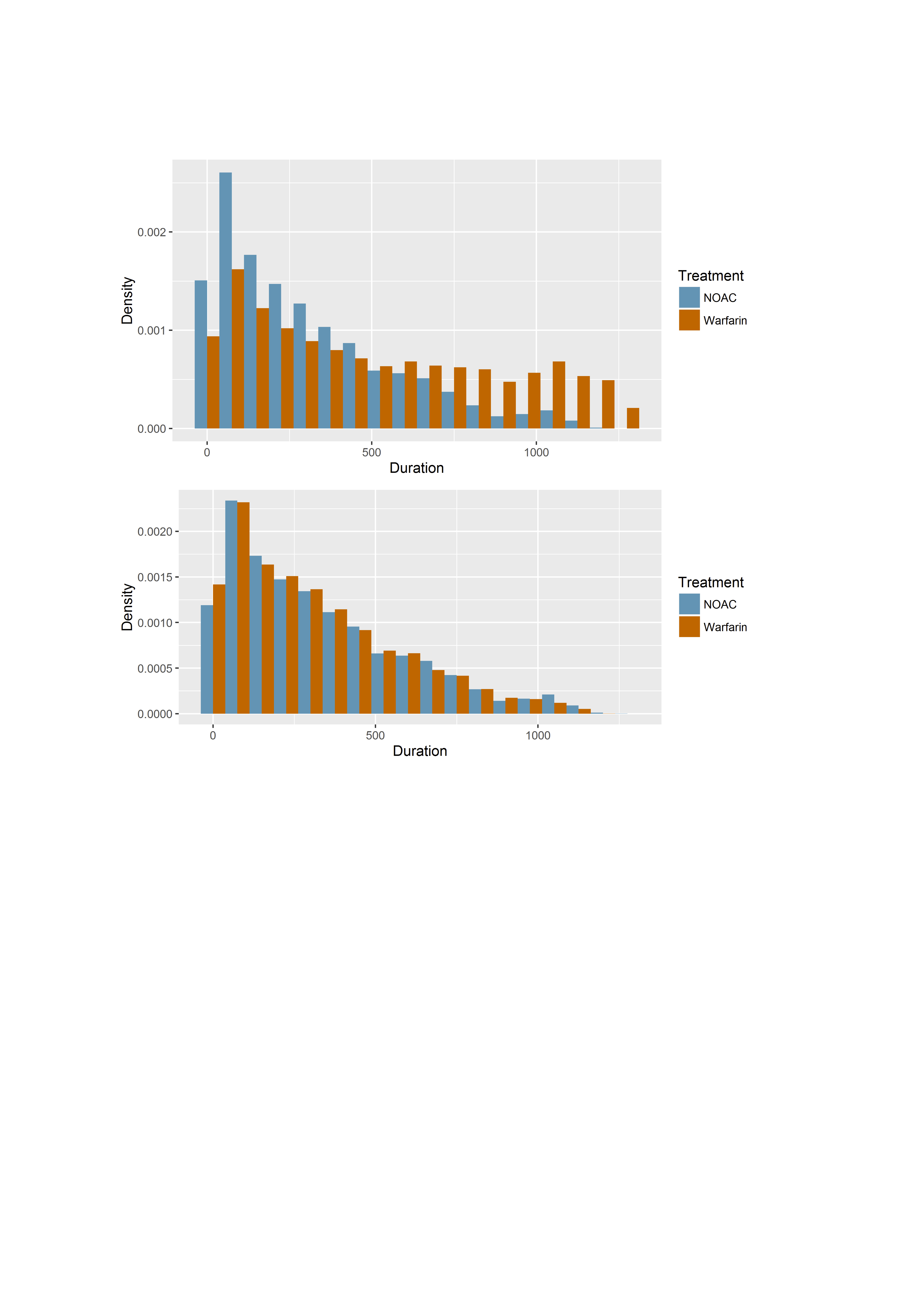

Supplement: S2 Fig — The unit on the x-axis is days. (TIFF) [file pone.0181000.s002.tiff]

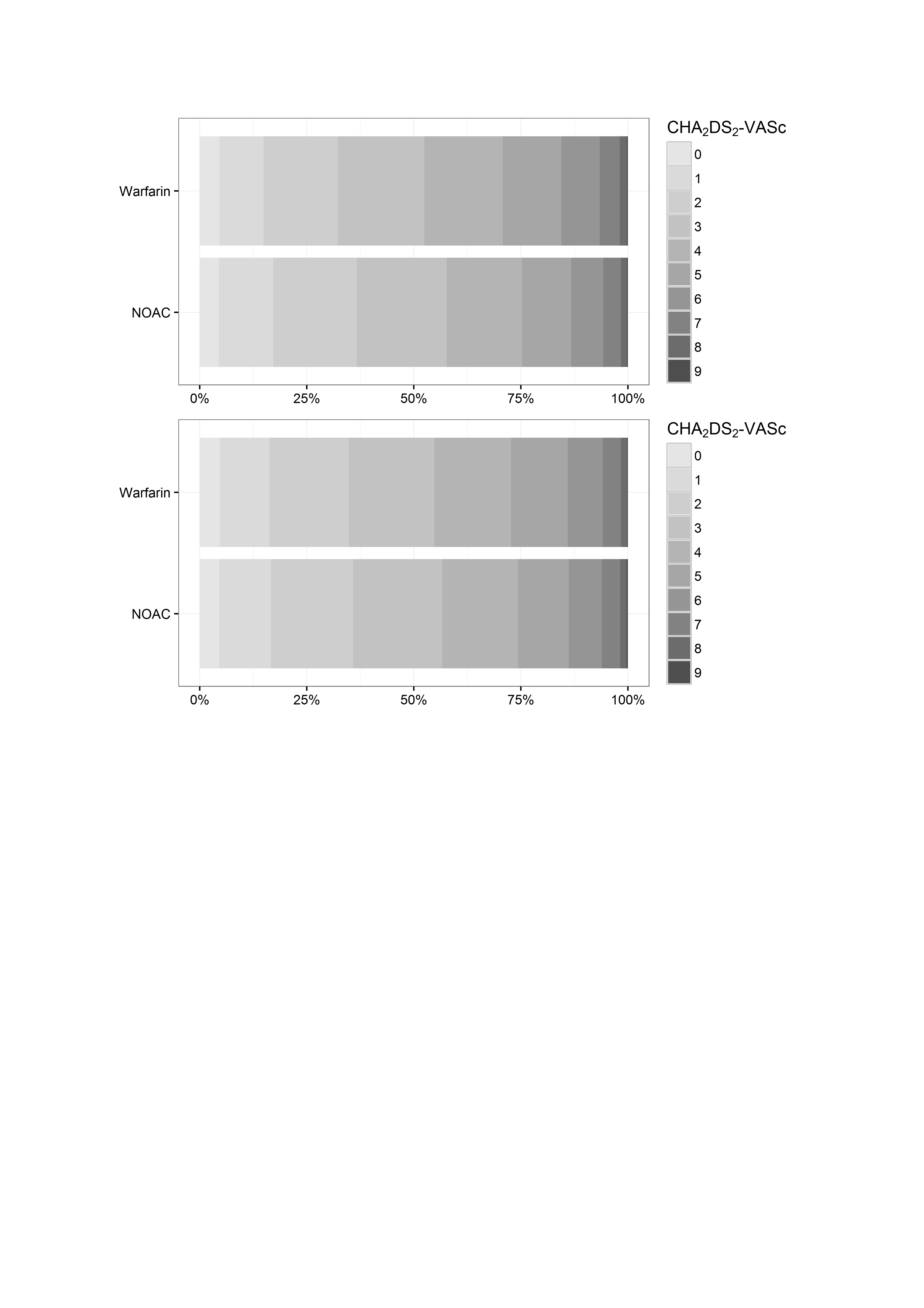

Supplement: S3 Fig — Upper diagram before, lower diagram after matching. Note that the score was not part of the matching, although components were. (TIFF) [file pone.0181000.s003.tiff]

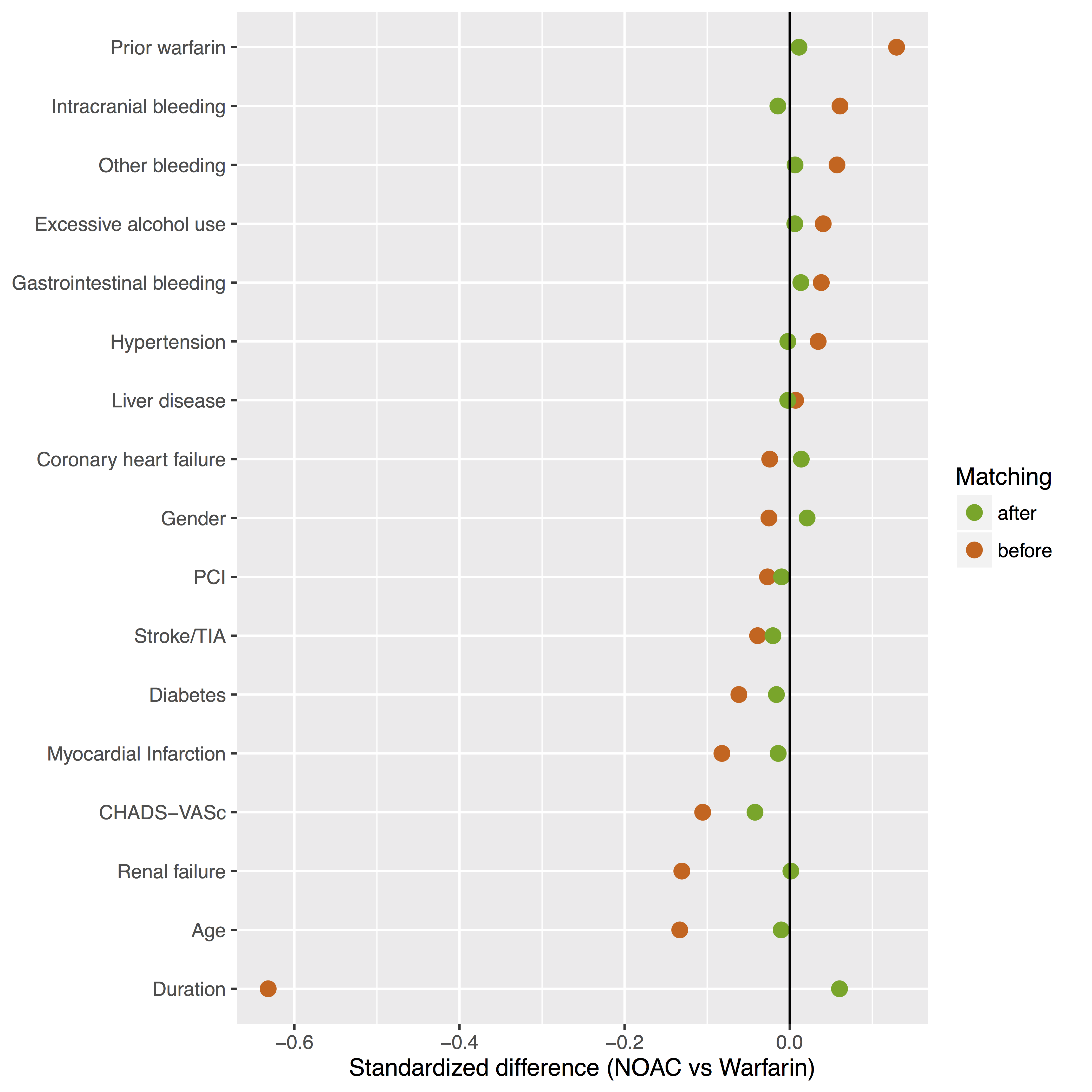

Supplement: S4 Fig — (TIFF) [file pone.0181000.s004.tiff]
