# Supplementary material for: Non-vitamin K oral anticoagulants are non-inferior for stroke prevention but cause fewer major bleedings than well-managed warfarin: A retrospective register study
Source: PLoS One. 2017 Jul 10;12(7):e0181000. doi: 10.1371/journal.pone.0181000 (PMC5507293; doi:10.1371/journal.pone.0181000)
Supplement: S1 Table — Data source is the Swedish National Patient Register (NPR). ICD-10 codes for diagnoses. Other codes are Swedish procedure codes. (DOCX) [file pone.0181000.s005.docx]

| Item in medical history | Criteria |
| --- | --- |
| History of fall | ≥2 occurrences of W00–W19 |
| Cancer | C00–C26, C30–C41, C43–C58, C60–C97 |
| Stroke | I60, I61, I63, I64, I69 |
| Transient ischaemic attack (TIA) | G45 (except G454) |
| Stroke or TIA | I63, I64, I69, G45 (except G454) |
| Hypertension | I10–I13, I15 |
| Congestive heart failure | I110, I130, I132, I50 |
| Diabetes | E10–14 |
| Myocardial infarction | I21, I252 |
| Ischaemic heart disease | I20–I23, I241, I248, I249, I251, I252, I255, I256, I258, I259 |
| Chronic obstructive pulmonary disease | J43, J44 |
| Anaemia | D50, D510, D513, D518, D519, D52, D53, D55, D560–562, D568, D569, D570–D572, D588, D589, D59–D64 |
| Major bleeding | D629, I60–I62, I850, I983, K250, K252, K254, K256, K260, K262, K264, K266, K270, K272, K274, K276, K280, K282, K284, K286, K625, K920, K922 |
| Gastrointestinal bleeding | I850, I983, K250, K252, K254, K256, K260, K262, K264, K266, K270, K272, K274, K276, K280, K282, K284, K286, K625, K920, K922 |
| Intracranial bleeding | I60–I62, S064–S066 |
| Cerebral haemorrhage | I60, I61 |
| Previous traumatic intracranial bleeding | S064–S066 |
| Renal failure | I120, I131, I132, N182–N185, N189, DR016, DR024, KAS00, KAS10, KAS20 |
| Excessive alcohol use | E244, F10, G312, G621, G721, I426, K292, K70, T51, Y90, Y91, K860, O354, P043, Q860, Z714 |
| Dementia | F00–F03 |
| Liver disease | K70–K77, JJB, JJC |
| Vascular disease | I21, I22, I252, I70–I73 |
| PCI | Z955 |
