# Supplementary material for: Non-vitamin K oral anticoagulants are non-inferior for stroke prevention but cause fewer major bleedings than well-managed warfarin: A retrospective register study
Source: PLoS One. 2017 Jul 10;12(7):e0181000. doi: 10.1371/journal.pone.0181000 (PMC5507293; doi:10.1371/journal.pone.0181000)
Supplement: S2 Table — Data source is the Swedish National Patient Register (NPR) except for RS: The Swedish Stroke Register (Riksstroke), or death noted in the Cause of Death register. (DOCX) [file pone.0181000.s006.docx]

| Outcome | Criteria |
| --- | --- |
| All-cause stroke and systemic embolism | NPR: I60, RS: I61, I63, I64, NPR: I74 |
| All-cause stroke | NPR: I60, RS: I61, I63, I64 |
| Ischaemic stroke | RS: I63 |
| Haemorrhagic stroke | NPR: I60, RS: I61 |
| Major bleeding | Any of intracranial, gastrointestinal, or other bleeding |
| Intracranial bleeding | NPR: I60, RS: I61, NPR: I62, S064–066 |
| Gastrointestinal bleeding | I850, I983, K250, K252, K254, K256, K260, K262, K264, K266, K270, K272, K274, K276, K280, K282, K284, K286, K625, K920–922 |
| Other bleeding | H113, H313, H356, H431, H450, H922, I312, J942, M250, N02, N501A, N938, N939, N950, R04, R319, R58, T810, D500, D508, D509, D629 |
| All-cause mortality | Presence in the Swedish cause of death register |
| Myocardial infarction | I21, I22 |
